# Procedural difficulty differences according to tumor location do not compromise the clinical outcome of laparoscopic complete mesocolic excision for colon cancer: a retrospective analysis

**DOI:** 10.18632/oncotarget.19780

**Published:** 2017-08-01

**Authors:** Min Ki Kim, In Kyu Lee, Bong-Hyeon Kye, Jun-Gi Kim

**Affiliations:** ^1^ Department of Surgery, Seoul St. Mary's Hospital, College of Medicine, The Catholic University of Korea, Seoul, Republic of Korea; ^2^ Department of Surgery, St. Vincent Hospital, College of Medicine, The Catholic University of Korea, Seoul, Republic of Korea

**Keywords:** laparoscopy, colonic neoplasms, survival, treatment outcome, colectomy

## Abstract

Laparoscopic colectomy procedures and their corresponding difficulty levels may vary depending on the tumor location within the colon, and a laparoscopic complete mesocolic excision (CME) with central vascular ligation (CVL) would require more proficiency than a conventional laparoscopic colectomy. We aimed to report our laparoscopic CME with CVL data and to investigate the clinical outcome differences of laparoscopic CME with CVL by various tumor sub-site locations. Prospectively collected clinical data of consecutive patients who received laparoscopic colectomy for primary colon cancer between April 1995 and December 2010 from single surgeon were retrospectively reviewed. All of the included surgery was performed on the basis of CME with CVL principle with no-touch isolation technique. Data were analyzed and compared among three groups; patients who received right or extended right hemicolectomy (group A, *n* = 142), transverse colectomy or left or extended left hemicolectomy (group B, *n* = 59), and sigmoidectomy or anterior resection (group C, *n* = 210). Female patients were more common in group A (53.5% vs. 37.3% vs. 39.5%, *p* = 0.020). Other baseline characteristics were comparable. Operative time was shorter in group C than the other groups (309.0 ± 74.7 vs. 324.3 ± 89.1 vs. 280.1 ± 93.1 min, *p* = 0.000). There was no significant difference among groups in perioperative complication and patient recovery. Five-year overall survival, disease-free survival and local recurrence rate showed no difference for a median follow up period of 73 (1–120) months. In conclusion, laparoscopic tumor-specific CME and CVL for colon cancer can be performed with comparable short- and long-term outcomes regardless of tumor sub-site location except for the operative time.

## INTRODUCTION

Studies [1, 2] that have assessed complete mesocolic excision (CME) with central vascular ligation (CVL) in colon cancer surgery have reported better oncological outcomes compared to those of conventional surgery. Mesocolic tissues containing lymphatics and vessels are the avenues for tumor spread, and hence the complete removal of mesocolic tissue must be performed meticulously along the correct plane without visceral fascial injury.

Laparoscopic surgery has been increasingly applied for colon cancer because several randomized trials [3–6] have indicated that this approach entails less pain, faster patient recovery and comparable oncological outcomes compared to conventional open surgery. However, previous articles regarding CME with CVL were mostly limited to open surgeries. Thus, whether this principle can be maintained in laparoscopic surgery has not been fully determined.

Additionally, procedures for colon cancer surgery can differ according to the tumor location. The colon exists in all quadrants of the abdominal cavity, and the main feeding vessel differs based on the tumor position. The short- and long-term outcomes can vary after cancer surgery depending on the procedure difficulty. Complication rates may increase in a difficult procedure, and the survival outcome may be impacted if an operation is conducted against the principle to avoid surgical difficulties. For this reason, when the concept of CME with CVL was not established, most studies comparing laparoscopic and open colorectal surgery excluded transverse and descending colon cancer, which are known to be difficult for the performance of laparoscopic surgery. Although CME with CVL has been accepted as a standard principle in colon cancer surgery, there are few data about whether laparoscopic CME with CVL is feasible for all lesions in the colon, or about its short- and long-term outcomes.

This study aimed to report on the short- and long-term outcomes of our laparoscopic CME with CVL surgery for colon cancer without excluding any colonic sub-sites and to determine whether the results of laparoscopic colon cancer surgery differed among specific procedures according to the tumor location.

## MATERIALS AND METHODS

### Study population

We retrospectively reviewed prospectively collected data from stage I–III primary colon adenocarcinoma patients who received curative resection from a single surgeon (J-G Kim) between April 1995 and December 2010. A total of 665 consecutive patients underwent colon cancer surgery from this particular surgeon during that period, of which 411 patients were included in the final analysis. We used the following exclusion criteria: open surgery, single-port laparoscopic or robotic surgery, palliative surgery, synchronous colon cancer, other intra-peritoneal cancer diagnosis within 5 years of colon cancer surgery, stage 0 or IV, emergency surgery, subtotal colectomy, or appendiceal cancer.

**Figure 1:**
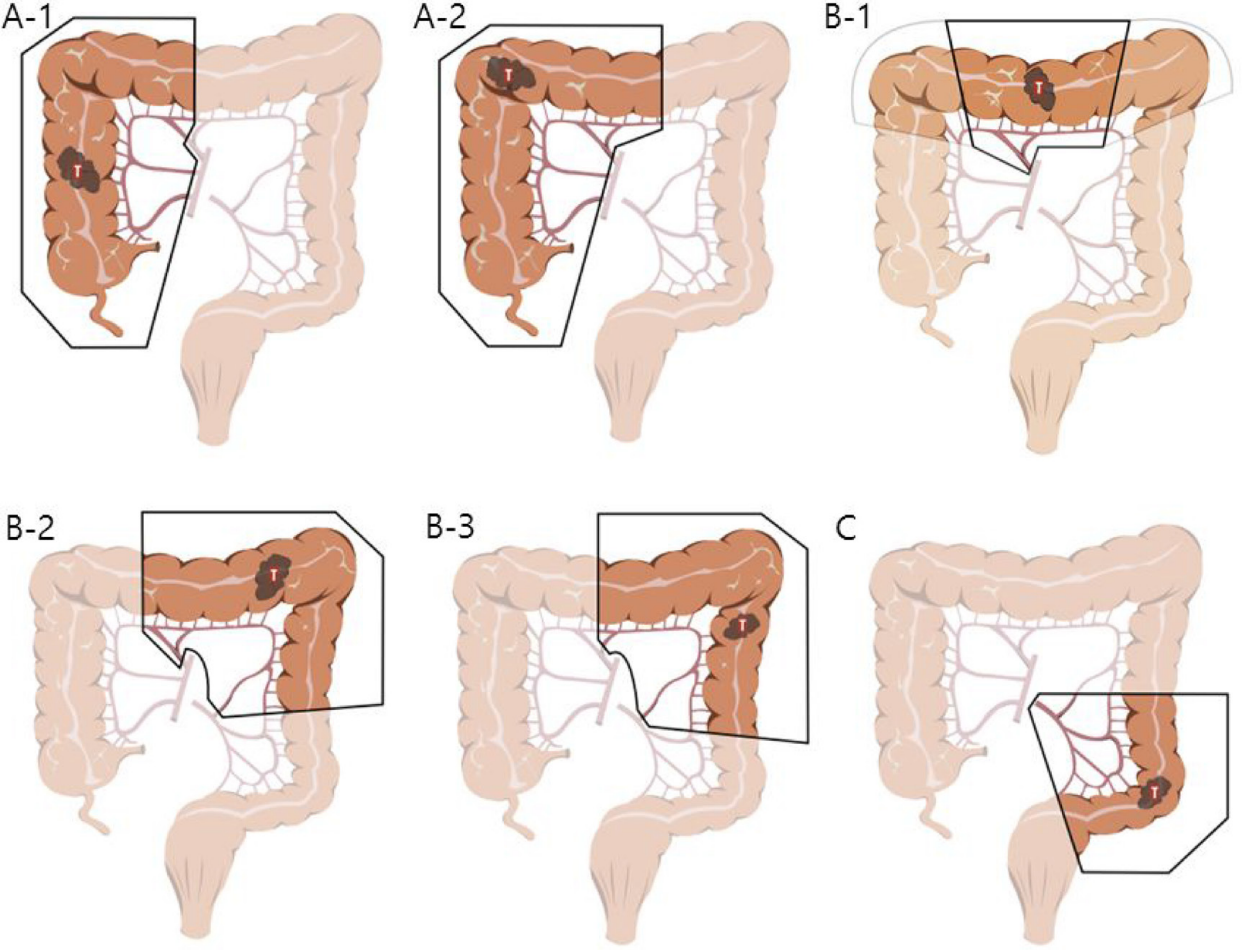
Illustrations describing specific procedures of three groups In the group (**A**) right hemicolectomy (A-1) and extended right hemicolectomy (A-2) were included. In the group (**B**) transverse colectomy (B-1, note that light line means only dissection, not resection), extended left hemicolectomy (B-2) and left hemicolectomy (B-3) were included. Sigmoid colectomy or anterior resection cases were included in the group (**C**).

The patients were subdivided according to the procedure they underwent as follows: right or extended right hemicolectomy (group A, *n* = 142), transverse colectomy or left or extended left hemicolectomy (group B, *n* = 59), and sigmoidectomy or anterior resection (group C, *n* = 210). This categorization was made based on the characteristics of the different surgical procedures for each area as follows (Figure 1).

For group A, right hemicolectomy or extended right hemicolectomy cases were included. Lymph node dissection (LND) was performed along the superior mesenteric pedicle, including its front side, with high ligation of the ileocolic vessels, middle colic vessels (for hepatic flexure and proximal transverse colon lesion), or right branch of the middle colic vessels (for lesions proximal to hepatic flexure colon). For group B, transverse colectomy, left hemicolectomy or extended left hemicolectomy cases were included. LND was performed on the origin site of the middle colic vessels (left branch of the middle colic vessels in case of left hemicolectomy) and to the origin site of the left colic artery for the complete removal of the mesocolon. Full splenic flexure mobilization was also required for all of the patients in this group. In contrast to the two groups above, group C required LND only around the inferior mesenteric artery (IMA). In other words, for group C, sigmoid colectomy or anterior resection cases were included. The requirement for splenic flexure mobilization was determined on a case-by-case basis. Preoperative informed consent was obtained from all patients, and this study was approved by the Institutional Review Board of the Catholic University of Korea.

### Perioperative management

A colonoscopic biopsy was used to confirm adenocarcinoma. The tumor location and clinical stage were judged by performing a colonoscopy, abdomino-pelvic computed tomography (CT) and/or barium enema. Mechanical bowel preparation and preoperative intravenous antibiotics were prescribed for all patients. Five-fluorouracil-based adjuvant chemotherapy was administered according to the postoperative pathology results and clinical judgment.

### Surgical procedures

**Figure 2:**
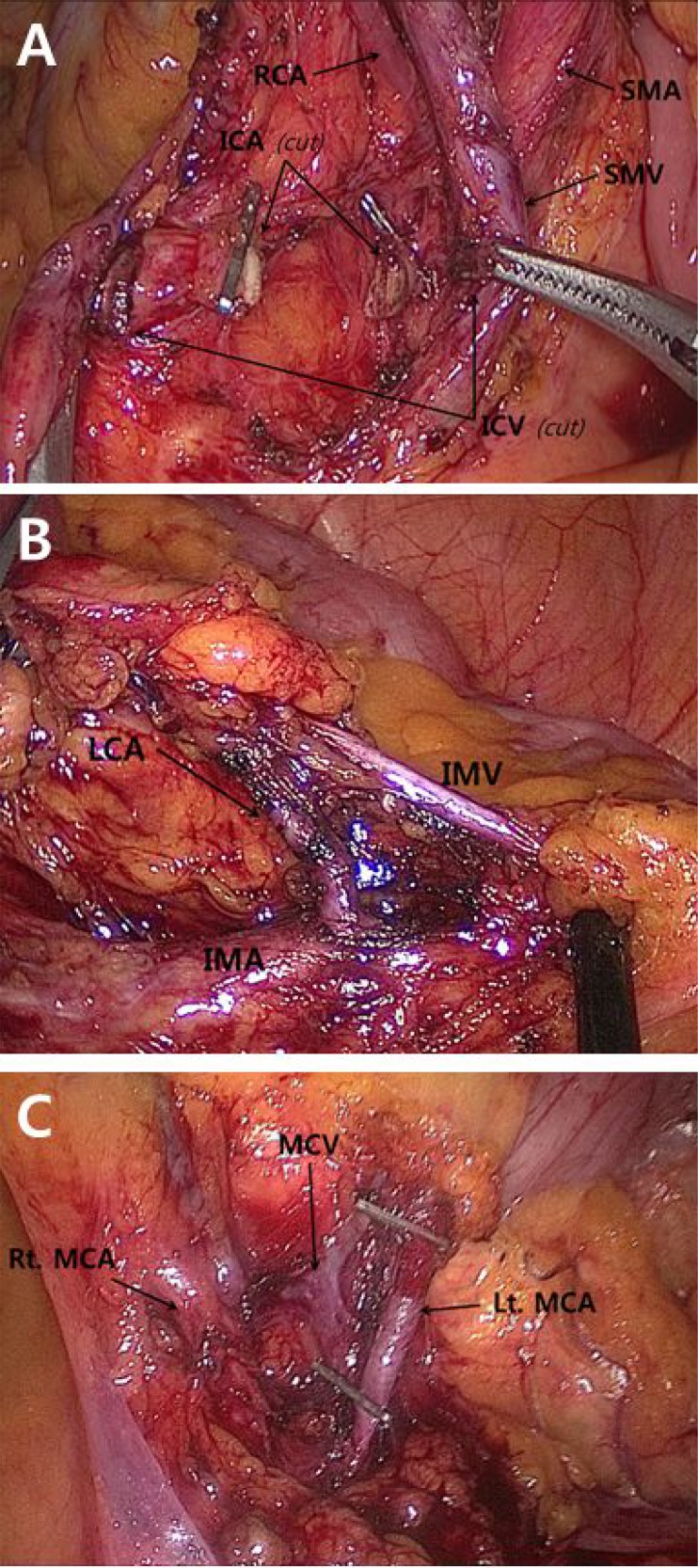
Intraoperative images of main colonic blood vessels Each vessels were strictly ligated and divided at their origins. Figure (**A**) was obtained from right hemicolectomy, and figure (**B**) and (**C**) were from left hemicolectomy. *SMA* superior mesenteric artery, *SMV* superior mesenteric vein, *ICA* ileocolic artery, *ICV* ileocolic vein, *RCA* right colic artery, *MCA* middle colic artery, *MCV* middle colic vein, *IMA* inferior mesenteric artery, *IMV* inferior mesenteric vein, *LCA* left colic artery.

**Figure 3:**
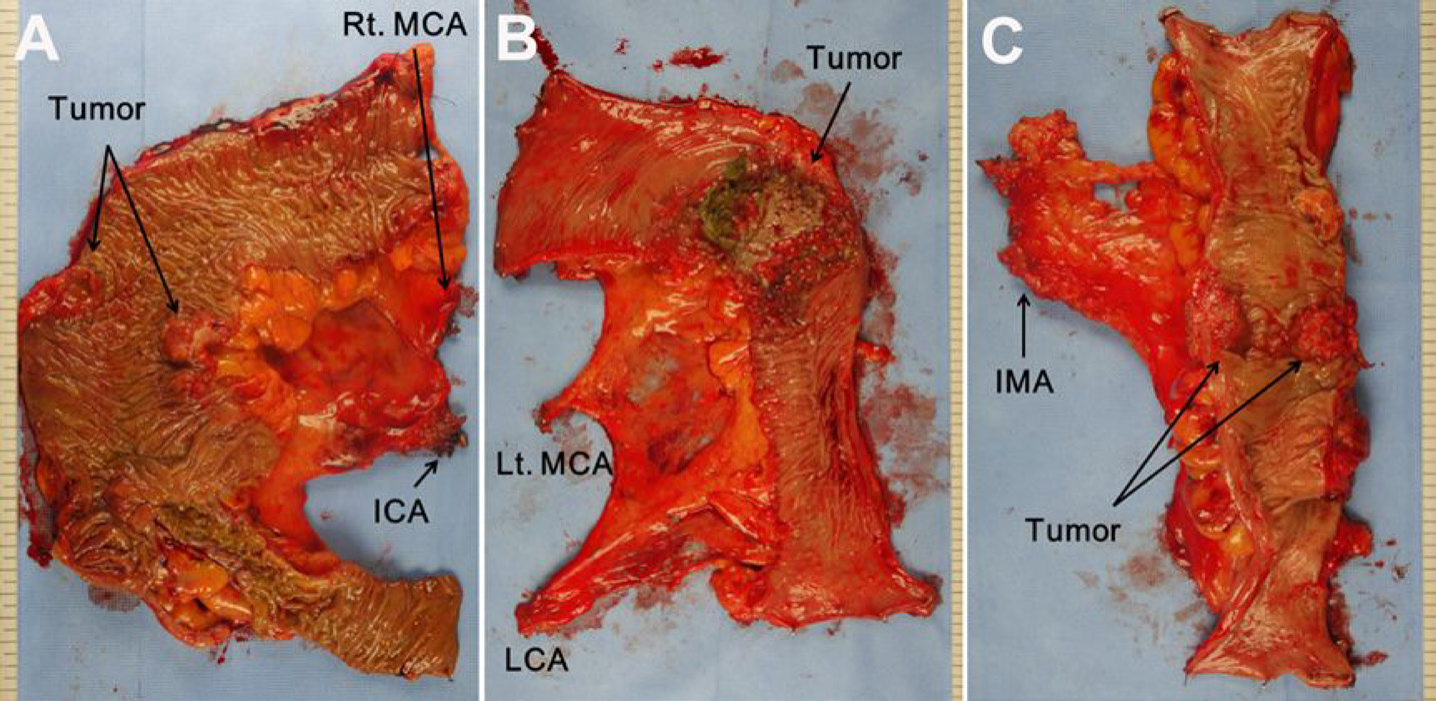
Postoperative fresh specimen of laparoscopic colon resection according to the tumor location (**A**) Right hemicolectomy. (**B**) Left hemicolectomy. (**C**) Anterior resection. MCA middle colic artery, ICA ileocolic artery, LCA left colic artery, IMA inferior mesenteric artery.

The surgical approach applied to all cases included dissecting the colon and mesocolon from the adjacent organs and tissues within the range of resection without injuring the visceral fascia layer to excise the tumor-bearing segment and maximize the LND by ligating the main artery at its origin. The surgeon attempted to secure 10 cm or more for the proximal and distal resection margins (over 5 cm distal margin for rectosigmoid lesions) (Figure 2). These procedures aligned with the basic concept of CME and CVL except that the gastroepiploic vessels were only meticulously dissected instead of routinely ligated even in the transverse colon lesion and a medial to lateral approach was used. The surgery in this study is different from original CME with CVL which was presented by Hohenberger et al. [1] in that it has more restricted standard for longitudinal margin, and differs from that of Japanese guideline [7] in that high ligation was performed regardless of clinical stage. Therefore, this can be called “tumor-specific CME” as in recent article [8]. Figure 3 presents examples of fresh specimens obtained from the surgical procedures in this article.

### Postoperative follow-up

A follow-up assessment was conducted every 3 months within 2 years of the surgery, biannually 2–5 years after the surgery, and annually 5 years postoperatively. The patient history, physical status, and carcinoembryonic antigen (CEA) levels were checked at each follow-up visit. An abdomino-pelvic CT was performed biannually, and a colonoscopy was performed annually. Chest CT or positron emission tomography was performed if clinically necessary. The last follow-up was performed in November 2015. Four patients were lost to follow-up and were excluded from the long-term survival analysis.

### Measured outcomes

The following variables were measured as a whole or compared among groups: basic patient characteristics, operative outcome, including intraoperative complications and postoperative complications (within 30 days after surgery or during the same admission period), patient recovery course, pathological characteristics, and long-term oncologic outcome (local recurrence rate, 5-year disease free survival [DFS] and 5-year overall survival rate [OS]). For surgical specimen morphology, the mesenteric margin was defined as the distance between the main feeding artery and the cancer mass.

### Statistical analysis

IBM SPSS ver. 18.0 (IBM Co., Armonk, NY, USA) was used for all statistical analyses. To compare the three groups, ANOVA (analysis of variance) test was performed for continuous data, and Fisher's exact test was used for categorical data. When there was a significant difference among groups, the Sceffe or Games-Howell method was adopted for inter-group analysis. The Kaplan-Meier method was performed for survival analysis, and the survival comparisons were performed with the log-rank test. Follow-up periods longer than 120 months were calculated at this time point. Statistical significance was defined as *P* < 0.05.

## RESULTS

### Patient characteristics and operative and post-operative management

Average age and body mass index (BMI) of total patients were 62.5 ± 11.6 years and 23.5 ± 3.0 kg/m^2^, respectively. No significant differences were detected in the basic characteristics, such as age, body mass index (BMI), American Society of Anesthesiologists (ASA) score, history of previous abdominal surgery, and preoperative CEA level. Synchronous intra-peritoneal organ resection and the rate of adjuvant chemotherapy did not significantly differ among groups (Table 1). More women were included in group A compared to the other groups (63.5% vs. 37.3% vs. 39.5%, *p* = 0.020).

**Table 1:** Patient characteristics and perioperative management

		A	B	C	*P* value
Age (years)	Mean ± S.D	63.5 ± 11.1	63.3 ± 10.2	61.7 ± 12.2	0.332
Gender	Male (%)	66 (46.5%)	37 (62.7%)	127 (60.5%)	0.020
BMI (kg/m2)		23.2 ± 2.9	24.1 ± 3.4	23.6 ±2.9	0.137
ASA					0.165
	1	97 (68.3%)	38 (64.4%)	159 (75.7%)	
	2	37 (26.1%)	20 (33.9%)	40 (19.0%)	
	3	8 (5.6%)	1 (1.7%)	10 (4.8%)	
	4	0 (0.0%)	0 (0.0%)	1 (0.5%)	
Previous abdominal surgery		26 (18.3%)	8 (13.6%)	28 (13.3%)	0.436
Synchronous resection of intraabdominal organ		7 (4.9%)	5 (8.5%)	26 (12.4%)	0.055
Preoperative CEA (ng/mL)		5.9 ± 12.4	4.1 ± 4.4	11.1 ± 70.0	0.630
Tumor site					0.000
	Cecum-HF colon	132 (93.0%)			
	Transverse colon	10 (7.0%)	6 (10.2%)		
	SF–Descending colon		53 (89.8%)		
	Sigmoid–Rectosigmoid colon			210 (100.0%)	
Adjuvant Chemotherapy		99 (70.2%)	41 (69.5%)	139 (66.2%)	0.732

BMI, body mass index; ASA, American Society of Anesthesiologists; CEA, carcinoembryonic antigen; HF, hepatic flexure; SF, splenic flexure.

### Pathological outcomes

Table 2 presents the pathological characteristics and surgical resection margins of the specimens. No differences were detected in the pathological stage according to the UICC 6th edition [9], T (tumor) and N (node) stage, histologic grade, or lymphatic/vascular/perineural invasion among groups. The proximal resection margin of group A was significantly longer (21.6 ± 12.0 vs. 12.6 ± 5.7 vs. 11.1 ± 4.0, *p* = 0.000), and the distal resection margin of group C was shorter than other groups (12.8 ± 5.5 vs. 13.0 ± 5.1 vs. 8.7 ± 4.4, *p* = 0.000). The number of harvested lymph nodes was significantly larger in group A (21.9 ± 13.3 vs. 12.7 ± 9.2 vs. 14.6 ± 8.9, *p* = 0.000).

**Table 2:** Pathologic characteristics and outcomes

		A	B	C	*P* value
AJCC Stage					0.197
	I	21 (14.8%)	7 (11.9%)	37 (17.6%)	
	II	73 (51.4%)	27 (45.8%)	82 (39.0%)	
	III	48 (33.8%)	25 (42.4%)	91 (43.3%)	
Pathologic T stage					0.142
	1	9 (6.3%)	3 (5.1%)	20 (9.5%)	
	2	18 (12.7%)	7 (11.9%)	33 (15.7%)	
	3	103 (72.5%)	49 (83.1%)	142 (67.6%)	
	4	12 (8.5%)	0 (0.0%)	15 (7.1%)	
Pathologic N stage					0.328
	0	93 (65.5%)	34 (57.6%)	118 (56.2%)	
	1	34 (23.9%)	16 (27.1%)	69 (32.9%)	
	2	15 (10.6%)	9 (15.3%)	23 (11.0%)	
Differentiation					0.076
	Well	31 (23.1%)	13 (23.6%)	37 (18.1%)	
	Moderately	97 (72.4%)	39 (70.9%)	165 (80.9%)	
	Poorly	6 (4.5%)	3 (5.5%)	2 (1.0%)	
Lymphatic invasion					0.441
	No	95 (70.9%)	40 (70.2%)	123 (64.4%)	
	Yes	39 (29.1%)	17 (29.8%)	68 (35.6%)	
Venous invasion					1.000
	No	133 (95.7%)	55 (96.5%)	190 (96.0%)	
	Yes	6 (4.3%)	2 (3.5%)	8 (4.0%)	
Perineural invasion					0.344
	No	121 (87.1%)	53 (93.0%)	168 (85.3%)	
	Yes	18 (12.9%)	4 (7.0%)	29 (14.7%)	
PRM (cm)	Mean ± S.D	21.6 ± 12.0	12.6 ± 5.7	11.1 ± 4.0	0.000
DRM (cm)	Mean ± S.D	12.8 ± 5.5	13.0 ± 5.1	8.7 ± 4.4	0.000
Mesenteric margin (cm)	Mean ± S.D	11.1 ± 3.7	12.0 ± 3.7	13.0 ± 3.0	0.000
Retrieved lymph nodes	Mean ± S.D	21.9 ± 13.3	12.7 ± 9.2	14.6 ± 8.9	0.000

PRM, proximal resection margin; DRM, distal resection margin.

### Intra- and postoperative outcomes

No differences were observed in the intraoperative complications or number of intraoperative transfusions, but the operative time was significantly shorter in group C than in the other groups (309.0 ± 74.7 vs. 327.2 ± 86.9 vs. 278.4 ± 93.2, *p* = 0.000) (Table 3). Markers for postoperative patient recovery, such as the time to pass flatus and return to diet, showed better trends in group C, but the differences were not clinically meaningful. The total conversion rate in our series was 4.1%, and no differences were observed among groups. The postoperative complication rate was 16.1%, but grade III or greater complications based on the Clavien-Dindo classification [10] scale were rare (1.9%). No significant differences were detected between groups. The leak rate was 1.2% and the reoperation rate was 1.9% without inter-group differences.

**Table 3:** Intra- and postoperative outcomes

	Total	A	B	C	*P* value
Operating time (min)		309.0 ± 74.7	327.2 ± 86.9	278.4 ± 93.2	0.000
Intraoperative transfusion (ml)		73.2 ± 221.5	33.9 ± 153.8	59.1 ± 296.0	0.607
Time to pass flatus (days)		3.2 ± 1.4	2.7 ± 1.2	2.4 ± 1.1	0.000
Return to diet (days)		4.3 ± 1.9	4.5 ± 1.5	4.1 ± 1.5	0.154
Postoperative hospital stay (days)		11.2 ± 4.4	12.0 ± 4.5	11.7 ± 6.2	0.572
Conversion	17 (4.1%)	8 (5.6%)	2 (3.4%)	7 (3.3%)	0.552
Intraoperative complications	26 (6.3%)	12 (8.5%)	4 (6.8%)	10 (4.8%)	0.334
Postoperative complications*	8 (1.9%)	1 (0.7%)	1 (1.7%)	6 (2.9%)	0.363

*Grade III or over in Clavien-Dindo classification.

### Long-term survival outcomes

**Figure 4:**
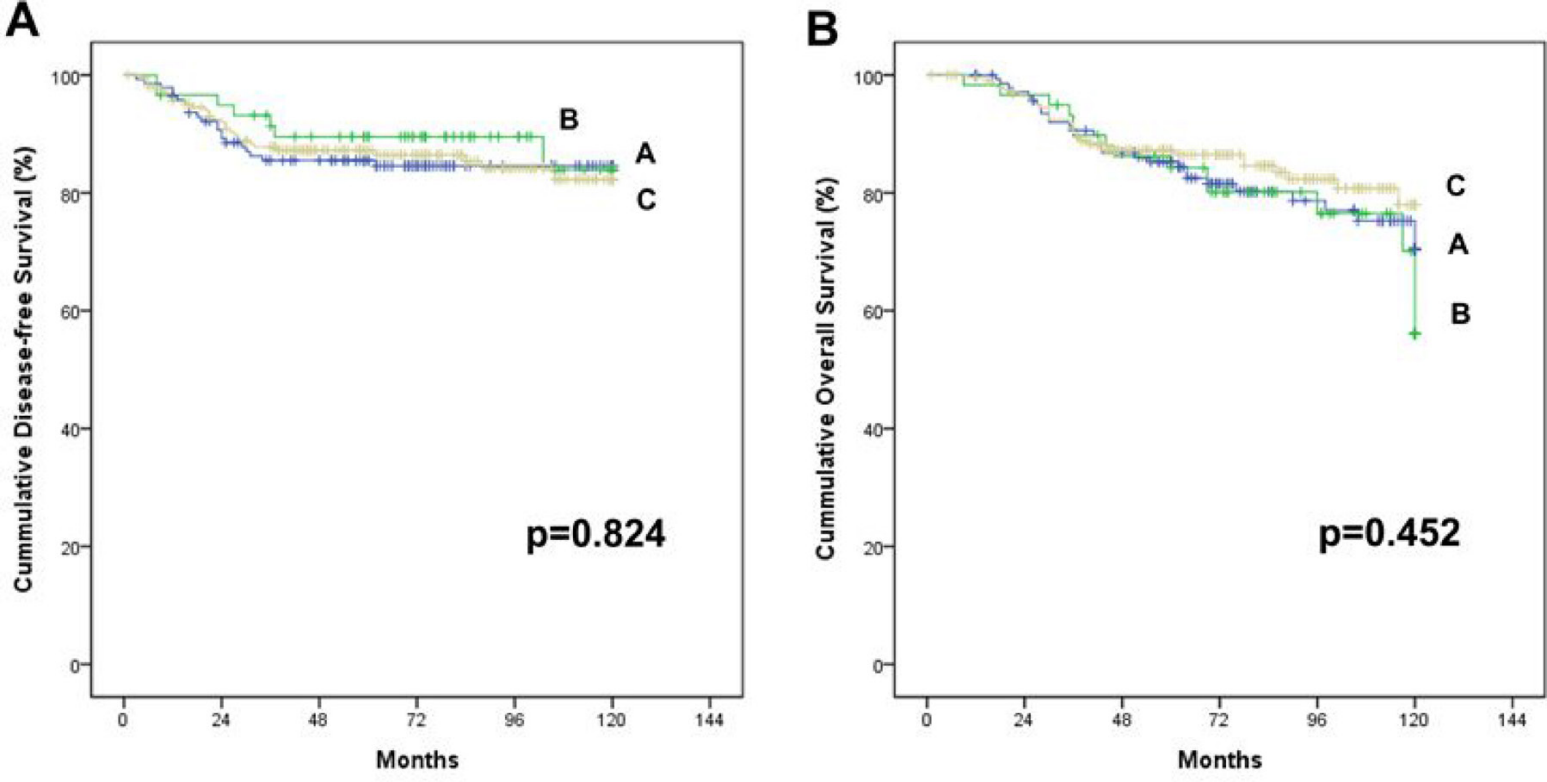
Cummulative survival of stage I–III colon cancer patients who received laparoscopic colectomy based on the complete mesocolic excision and central vascular ligation principle with no touch isolation technique There is no significant difference among the three groups; A, B and C. (**A**) Disease free survival rate (at 5 years, 85.5%, 89.5% and 87.2%, *p* = 0.824). (**B**) Overall survival rate (at 5 years, 85.3%, 84.3% and 87.2%, *p* = 0.452)

The median follow-up periods were 73 (1–120) months. The five-year DFS and 5-year OS were 89.9% and 86.0%, respectively, for the whole patients, and 79.3% and 74.1%, respectively for stage III. No survival differences were found among groups (log rank test, *p* = 0.824 for DFS, *p* = 0.452 for OS; Figure 4). Table 4 shows DFS and OS by stage, which did not significantly differ. Sixteen cases (3.9%) revealed local recurrence without inter-group differences (4.2% vs. 5.1% vs. 3.3%, *p* = 0.594).

**Table 4:** Survival outcomes of Stage I–III colon cancer patients that underwent laparoscopic CME and CVL

		Total	A	B	C	*p*-value
local recurrence		16 (3.9%)	6 (4.2%)	3 (5.1%)	7 (3.3%)	0.594
	stage I	96.5%				
5yr-DFS (%)	stage II	92.1%	88.9%	96.3%	93.6%	0.696
	stage III	77.7%	74.1%	83.4%	78.1%	0.668
	stage I	93.2%				
5yr-OS (%)	stage II	89.6%	87.5%	92.3%	90.7%	0.486
	stage III	79.3%	75.6%	74.8%	82.8%	0.360

DFS, disease-free survival; OS, overall survival.

## DISCUSSION

Our study showed that surgical dissection that incorporated the principle of mesenteric dissection with embryological planes and central vascular ligation could be performed laparoscopically for any major colon procedure without significant differences. Difficult laparoscopic procedures, such as splenic flexure mobilization and lymph node dissection along the superior mesenteric vein (SMV), were successfully performed with no increase in complications. This approach was associated with good long-term outcomes that included a 3.9% local recurrence rate in all patients and a 77.7% DFS rate and 79.3% OS rate in stage III patients.

The oncologic principle of surgical dissection that follows the embryological/anatomical planes has been emphasized in previous articles [11, 12]. Bokey et al. [11] showed better oncologic outcomes with anatomical resection of colon cancer. Maximizing the lymph node harvest has been considered another important principle in colon cancer surgery [7, 13]. However, these principles have not been universally accepted by surgeons to date. Hohenberger et al. integrated these principles and named them CME and CVL. They demonstrated that their procedure increased the survival rate based on an analysis of their laparotomy data for colon cancer. The surgeon in this study has followed the principle since 1984 and has continued to maintain the principle even after applying laparoscopy to his colorectal surgery in 1995.

The surgeon in this study has secured one more surgical principle in his surgery: the medial to lateral approach, which is a major procedure of the “no-touch isolation technique”. This approach is based on the concept that a surgeon must handle the cancer-bearing segment in the last step after ligating the associated vessels and dissecting the lymph nodes to minimize possible tumor scattering during manipulation of the cancer. This principle, which was denominated by Turnbull et al. [14], has been emphasized for a long time. Turnbull et al. reported a difference in survival outcomes of approximately two-fold from securing this principle for advanced colon cancer in their retrospective study. However, the medial to lateral approach has not been widely adopted in conventional open surgery because its impact on survival has not been determined [15, 16] and this approach is somewhat difficult to perform. The introduction of laparoscopy has made the medial to lateral approach more feasible. Vessels and adjacent structures can be fully identified, and more meticulous manipulation is possible with laparoscopy. Notably, an old conventional principle was made restorable by a new technique.

Laparoscopic colon cancer surgery is associated with lower pain levels, faster recovery and reduced short-term mortality rates without compromising long-term survival compared to conventional open surgery [3–6]. Hence, patients can benefit from laparoscopic surgical approaches and receive the oncologic benefits of CME and CVL if laparoscopic CME and CVL are applied. However, a meta-analysis which gathered 7 case-controled trials and 1 randomized controlled trial comparing laparoscopic and open CME with CVL surgery was published recently, and it included only one paper with data on all segments of the colon (written in Chinese only) [17]. There are still scarce data about long-term results of laparoscopic CME with CVL that includes all segments of the colon, or on outcome comparisons according to the tumor sub-site location.

Transverse colectomy and left hemicolectomy are difficult to perform laparoscopically because they require not only LND around the SMV without damaging vulnerable organs, such as the pancreas and duodenum, but also the full mobilization of splenic flexure [18, 19]. However, no differences in complications or survival rates were observed when the three groups were compared, even with the CME and CVL principle.

The only difference among groups was the operative time. Laparoscopic surgery of the ascending colon has often been viewed as relatively easy; however, no significant difference in the operative time was observed between group A and B, and the operative time was significantly shorter for group C than for the other groups. This result may have occurred because cancers in the former two groups usually need LND for a dual blood supply (ileocolic and middle colic or middle colic and left colic), whereas cancers in group C need only LND for a single blood supply (inferior mesenteric).

The resection margin was longer and the lymph node harvest was greater in the group A, which could be attributable to anatomic differences rather than the surgical technique. Previous reports [20, 21] also showed greater amounts of lymph node harvest in ascending colon lesions that could be attributable to several factors. Generally, unlike lesions between the transverse and sigmoid colon, the right side colon has larger tumors and more microsatellite instability [22], which are known to be associated with higher lymph node harvest numbers [23].

Several studies have discussed the differences between right and left colectomies and have reported inconsistent results for short-term complications. Some studies [24, 25] concluded that right colectomies were associated with better outcomes, whereas others [26] reported that left colectomies were superior. Another study [27] found no difference in the complication rates when right and left colectomies were compared. However, the different results could be biased due to various situations, such as the surgical technique, the learning curve of each technique, whether the principles of oncologic surgery were adhered to regardless of the lesion location, whether the studied lesion was benign or malignant, and whether the operation was performed by open or laparoscopic surgery. Our data indirectly suggest that similar results can be obtained by investing more time when performing the oncologic surgery even though different locations can have different degrees of difficulty in colon cancer.

Some authors have reported different survival outcomes according to the tumor subsite in colon cancer. Bhangu et al. [28] analyzed Surveillance, Epidemiology, and End Results Program (SEER) data calculating the adjusted hazard ratio and concluded that sigmoid colon cancer had superior survival rates compared with colon cancer in other areas. The same results were shown in a study of a California registry [20] that used a multivariate analysis and adjusted for other factors. Sjo et al. [29] reported that tumors located in the transverse colon, splenic flexure, and descending colon had a poor prognosis compared to tumors in other locations.

Additional studies are needed to validate this hypothesis because surgical quality was not included in the adjusting variables. As our study reveals, laparoscopic right hemicolectomy is as difficult as transverse colectomy or left hemicolectomy. The rate of complications can increase depending on the difficulty of the surgery, and surgeons may opt to perform a “safer” and “less radical” surgery to avoid complications. The duodenum and pancreas are nearby, and lymph node dissection along the SMV is necessary for colon cancers that are located between the ascending and descending colon. As mentioned above, when the case involves LND around critical vessels and vulnerable organs, the principle of CME and CVL may not be maintained due to the operative risk and difficulty, which can contribute to inferior survival outcomes compared to sigmoid colon lesions. Some studies have reported a tendency for reduced mesocolic plane security in the right-sided colon regardless of whether the operation is performed with the CME and CVL principle [30, 31]. Additional analyses are needed to determine whether the survival outcome differs among colonic subsite locations due to other biological factors.

The results of our study have implications for colorectal surgical training. Currently, minimal invasive surgery is preferable; however, maintaining consistency with the same principles as open surgery is important. Since the sigmoid colon can relatively easily adhere to the principle of CME and CVL regarding the operation time, a training program for laparoscopic colon cancer surgery is recommended based on our results. For lesions between the ascending and descending colon, laparoscopic surgery must be applied after accumulating sufficient anatomical knowledge and laparoscopic techniques by performing surgery for benign disease in these areas or surgery for malignant disease in the sigmoid colon.

Our study had the following limitations. First, this study was a retrospective study that analyzed a single surgeon's data. Hence, the analysis contains selection bias and may not be fully generalizable. Additional research is needed to determine whether laparoscopic application of CME and CVL can provide similar results for Western patients, who are usually more obese than Korean patients.

Second, bias could have originated from the pathologists. For example, radical resections were performed, and a corresponding relatively high survival outcome was shown in our study even though the number of harvested lymph nodes was smaller compared to other studies. According to some authors [32, 33] the number of harvested lymph nodes can differ depending on the pathologist or pathological examination quality. All the surgeries included in this study were performed in two hospitals. Significantly more lymph nodes were harvested from the latter (15.1 ± 9.5 vs. 25.3 ± 14.8, *p* = 0.000 by student's *t* test). The difference in the number of harvested lymph nodes between the two hospitals suggested that the pathologists from the former hospital were less cautious about the LN harvest. The importance of a full examination for even benign lymph nodes by pathologists has been highlighted since the middle of our study period in Korea. However, this limitation could not have influenced the results because we compared different procedures performed during the same period.

These limitations warrant further studies to confirm the results of this study.

## CONCLUSIONS

From the perspective of providing patients with improved oncologic impact with the strong advantages of laparoscopic surgery, incorporating CME and CVL with no-touch isolation through laparoscopy for any location could be considered when performing colon cancer surgery.

## Abbreviations

CME: Complete mesocolic excision
CVL: central vascular ligation
LND: lymph node dissection
CT: computed tomography
CEA: carcinoembryonic antigen
DFS: disease free survival
OS: overall survival
BMI: body mass index
ASA: American Society of Anesthesiologists
SMV: superior mesenteric vein
